# Alveolar Bone Preservation Using a Combination of Nanocrystalline Hydroxyapatite and Injectable Platelet-Rich Fibrin: A Study in Rats

**DOI:** 10.3390/cimb45070377

**Published:** 2023-07-17

**Authors:** Andries Pascawinata, Gusti Revilla, Roni Eka Sahputra, Syukri Arief

**Affiliations:** 1 Doctoral Student of Biomedical, Faculty of Medicine, Andalas University, Padang 25163, Indonesia; andriespascawinata@fkg.unbrah.ac.id; 2 Department of Anatomy, Faculty of Medicine, Andalas University, Padang 25163, Indonesia; 3 Department of Surgery, Orthopaedic Division, Faculty of Medicine, Andalas University, Padang 25163, Indonesia; dekanat@med.unand.ac.id; 4 Department of Chemistry, Faculty of Mathematics and Natural Sciences, Andalas University, Padang 25163, Indonesia; kimia@sci.unand.ac.id

**Keywords:** nanocrystalline hydroxyapatite, injectable platelet-rich fibrin, TRAP, ALP, OCN, new bone, post-extraction healing

## Abstract

Alveolar bone resorption is a post-extraction complication wherein there is a reduction in the dimensions and quality of the alveolar bone. This study aimed to examine the effects of implantation of a combination of nanocrystalline hydroxyapatite (nHA) and injectable platelet-rich fibrin (IPRF) on the expression of tartrate-resistant acid phosphatase (TRAP), alkaline phosphatase (ALP), osteocalcin (OCN), and new bone formation. A total of 32 male rats had their upper right incisors extracted under general anesthesia and were then divided into a control group, nHA group, IPRF group, and nHA-IPRF group. Decapitation was carried out on day 14 and day 28 in each group and the jaws of each rat were subjected to immunohistochemical and histological analysis. The results showed a decrease in TRAP expression in the nHA-IPRF group compared with the control group on day 14 (*p* = 0.074) and day 28 (*p* = 0.017). The study also showed an increase in ALP and OCN in the HA-IPRF group on day 14 and day 28 compared with the control group. New bone formation suggested a significant increase in the nHA-IPRF group compared with the control group on day 14 (*p* = 0.001) and day 28 (*p* = 0.001). nHA-IPRF implantation can suppress alveolar bone resorption, which is indicated by decreased TRAP expression, and it can increase bone growth, as indicated by increased expression of ALP, OCN, and new bone formation.

## 1. Introduction

Alveolar bone is an important part of the maxillofacial bone, which supports the teeth. Alveolar bone loss can occur because of various local or systemic factors [1]. Morphological changes in alveolar bone have been known and explained by a number of pre-clinical and clinical studies, where preserving alveolar bone is one of the ways to prevent the changes [2,3,4,5]. Alveolar bone preservation aims to minimize bone loss and changes in alveolar bone dimensions after tooth extraction. The success of this procedure will support the rehabilitation process during the dental implants, which must be placed in the right vertical and horizontal dimensions on the alveolar bone [6,7]. An accurate procedure will ensure strong and durable dental implants in the oral cavity. Aside from preventing excessive resorption of alveolar bone, alveolar bone preservation using bone graft can also increase new bone growth and accelerate the healing process [7,8].

Various studies have tried to find the ideal bone graft [9]. Autograft remains the gold standard because it provides cells and alive tissues and does not cause immunogenic reactions from the host. Harvesting donor organs, on the other hand, requires additional surgery that will increase the risk and morbidity in patients. Alternatively, bone grafts can also be taken not only from other individuals or allografts, but also from different species or xenografts. The two types of bone grafts are advantageous because they can be produced abundantly, but have problems in disease transfer and immunogenic reactions [10]. At present, synthetic bone graft or alloplastic bone graft is being developed as the alternative bone grafts [11]. Alloplastic bone graft is a synthetic material that contains essential chemical components of natural bone such as calcium and phosphate. This bone graft is advantageous because it is a standardized material and prevents from the risk of infectious diseases compared with allograft and xenograft. Furthermore, it has biological stability and volume maintenance that allows cell infiltration and bone remodeling [12].

Nanocrystalline hydroxyapatite (nHA) is a synthetic material made from natural or artificial sources, which resembles natural hydroxyapatite in bone better than commercial hydroxyapatite. Besides, nHA is also known to have a close contact with surrounding tissues and is bioresorbable, surface free energy, and high binding energy [11,13]. nHA is known to have osteoconductive properties, which work as a scaffold for new bone growth. Despite a great deal of relative satisfaction with the use of nHA, several studies have shown the limitations of the osteoinductive properties of nHA and, thus far, no bone graft material has been found to have biological properties as good as autograft [11,14,15,16]. Therefore, researchers tried to combine nHA with various materials, such as injectable platelet-rich fibrin (IPRF), to improve its osteoinductive abilities. IPRF is a platelet concentrate obtained autologously or from blood from its own host centrifuged at a low speed. IPRF is rich in cytokines and growth factors that can increase cell proliferation and differentiation, promote angiogenesis, act as a matrix for tissue growth, and regulate inflammatory and anti-infective reactions. Growth factors from IPRF such as platelet-derived growth factor (PDGF), transforming growth factor (TGF-β), and insulin growth factor (IGF) are essential in bone healing and regeneration [9].

Healing in the socket following tooth extraction consists of several phases including the hemostasis and coagulation, inflammatory, proliferation, and modeling and remodeling phases, where all of the phases can overlap each other [3]. Some biomarkers secreted during the bone healing process include tartrate-resistant acid phosphatase (TRAP), which plays a role during bone resorption, as well as alkaline phosphatase (ALP) and other noncollagen extracellular bone matrix proteins, such as osteocalcine (OCN), which contributes to osteogenesis [17]. Based on the above background, the author is interested in examining the effects of combined nanocrystalline hydroxyapatite and IPRF implantation on the expressions of TRAP, ALP, OCN, and new bone formation on post-extraction bone healing in wistar rats.

## 2. Materials and Methods

### 2.1. Materials

#### Hydroxyapatite

**Figure 1:**
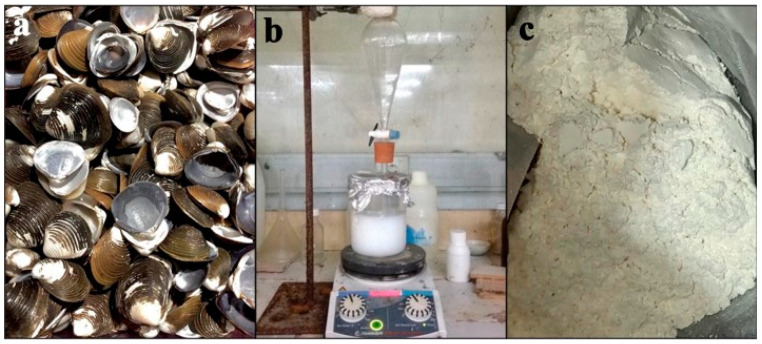
(**a**). Pensi shells, (**b**) hydroxyapatite synthesis, and (**c**) as-synthesized hydroxyapatite powder after calcination.

CaO was produced and taken from the pensi shells as a calcium precursor (*Corbicula moltkiana*). The pensi shells were first dried, cleaned with tap water, and then ground into a coarse powder. To produce CaO, the powder was calcined for five hours at 900 °C. HNO_3_, NH_4_OH, and (NH4)_2_HPO_4_ used were of analytical grade from Merck (Sigma-Aldrich, Burlington, MA, USA). In Figure 1, pensi shell images are shown (Figure 1a).

### 2.2. Hydroxyapatite Synthesis

**Figure 2:**
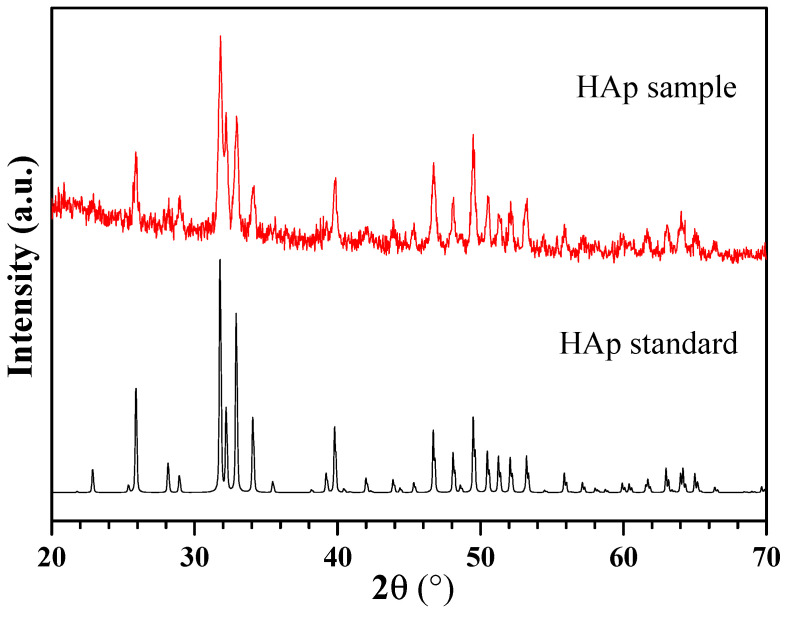
XRD pattern of hydroxyapatite.

**Figure 3:**
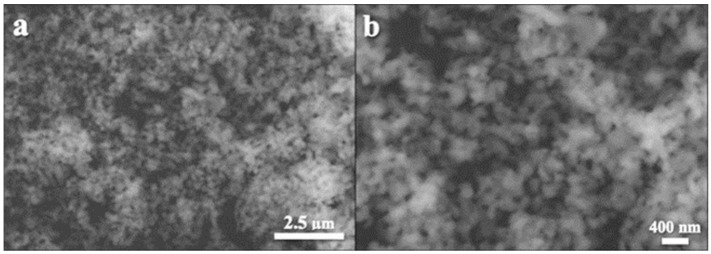
SEM images of hydroxyapatite sample at (**a**) 10,000× and (**b**) 20,000× magnification.

Based on the method described by Azis et al. and Labanni et al., hydroxyapatite (HAp) was synthesized in the following steps: 75 mL of 2 M HNO_3_ and 4.2 g of CaO were added, followed by 15 min of 500 rpm stirring at 85 °C. The remedy was then filtered. The filtered solution was mixed at 500 rpm at 110 °C with the addition of a total volume of 250 mL of a 0.18 M (NH4)_2_HPO_4_ solution. The mixture’s pH was raised to 11 during the reaction by the addition of NH_4_OH. All of the (NH4)_2_HPO_4_ was added and the mixture was then stirred continuously for five hours at 100 °C. After being left for 24 h, the mixture was filtered to form a gel. The gel was dried in an oven set to 110 °C for four hours [18,19]. To create hydroxyapatite powder, the obtained solid was ground into powder and then heated to 800 °C for three hours. X-ray diffraction (XRD) (XPERT PRO Pananalytical PW30/40) and scanning electron microscopy (SEM) (HITACHI S-3400N) were used to perform the characterization. XRD analysis showed that the material was high crystallinity hydroxyapatite (Figure 2). SEM analysis showed that the material contains of evenly distributed particles which have sphere-like shape and mean diameter of about 100 nm (Figure 3).

#### 2.2.1. Injectable Platelet-Rich Fibrin (IPRF)

The rat blood was taken before tooth extraction. General anesthesia was performed using ketamine and xylazine, and then 1 mL of blood was taken from the periorbital vein. Blood samples of each rat were centrifuged with a centrifugation device for 3 min at a speed of 700 rpm, after which IPRF was separated from the blood plasma. The IPRF was made based on the protocol in the study of Karde, et al. (2020) [20].

#### 2.2.2. Combination of nHA and IPRF

**Figure 4:**
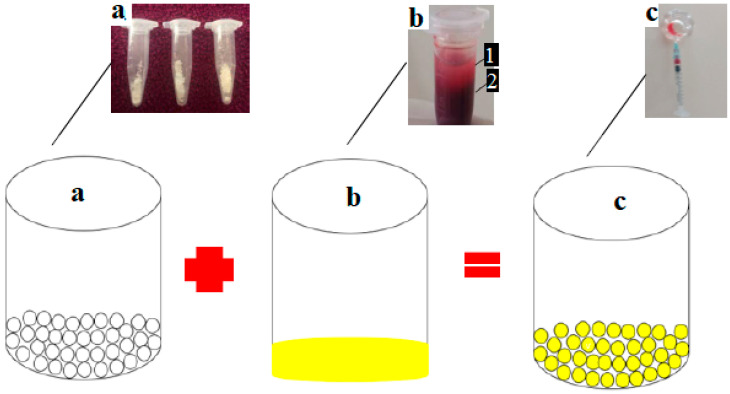
(**a**). Nanocrystalline hydroxyopathy: (**b**) (1) IPRF and (2) blood; (**c**) combination of HA and IPRF.

The combination of nHA in the form of 20 mg powder was mixed into 0.2 mL IPRF to form a suspension (Figure 4).

### 2.3. Preparation of Experimental Animals

Referring to the authorization letter No. 338/UN.16.2/KEP-FK/2021, the use of experimental animals has been allowed by the research ethics committee of the faculty of medicine at Andalas University in Padang, Indonesia. Group I (control day 14), Group II (control day 28), Group III (IPRF day 14), Group IV (IPRF day 28), Group V (HA day 14), Group VI (HA day 28), Group VII (HA-IPRF day 14), and 32 male rats aged more or less 2 months, weighing ±200 g, were divided into eight groups, each of which comprised four rats. The rats were then given food and water ad libitum.

### 2.4. Anesthesia and Surgical Procedures

**Figure 5:**
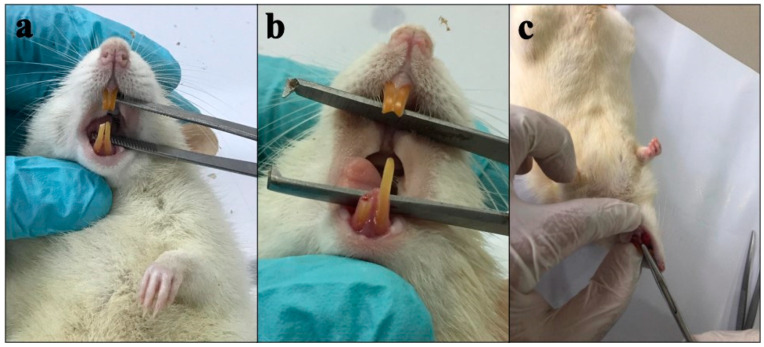
Lower incisor in rat (**a**) before cutting and (**b**) after cutting to the gingival margin, and (**c**) rat tooth extraction.

Ketamine and xylazine were used to perform general anesthesia. The method used to extract the teeth follows the technique described by Rakhmatia et al. [21]. Before the tooth extraction procedure, the right lower central incisor was sliced every three days up to twice in a row. The retention of the periodontal ligament was cut away at the gingival edge using a diamond disk bur to make tooth extraction easier. After cutting the teeth, the right lower central incisor was extracted in a horizontal direction along the tooth axis in the third phase, and the tooth was gently withdrawn using a needle holder with a controlled movement (Figure 5).

Euthanasia was carried out by administering a lethal dose of anesthetics. The rats’ right lower jaw was taken and placed in a solution of formalin buffer fixation for histological preparations. The post-extraction tooth socket was filled with IPRF, nHA, or nHA-IPRF in the treatment group using a micropipet, whereas the post-extraction tooth socket was kept unfilled in the control group. Following tooth extraction, rats received 0.3 mL each of the painkiller novalgin and the antibiotic gentamicin for 3 days. Four rats were applied to the control, IPRF, nHA, and nHA-IPRF groups on day 14 and 28, respectively. A deadly dose of anesthetics was used to induce decapitation.

### 2.5. Histological and Immunohistochemistry Analysis

The preparation was made through the process of decalcification, dehydration, clearing, paraffin infiltration, embedding, and 5 μm thick cutting with a transverse direction parallel to the sagittal plane using microtomes. In immunohistochemical examination, hydration and incubation were performed with rat primary polyclonal antibodies of TRAP (Fine biotech), ALP (ABclonal technology), or OCN (Fine biotech) overnight. Immunoreactivity of TRAP, ALP, and OCN was detected and visualized using secondary antibodies at room temperature for 1 h after washing. After reacting with 3.3′-diaminobenzidine, it turned brown as the staining signal. The specimen was observed using a microscope (Olympus Cx33) and camera (Sigma). To assess the formation of new bone in histological preparations, tissue slices were stained with hematoxylin/eosin. All observations were taken at three different locations representing the proximal, middle, and distal parts of the dental socket.

The measurement method used immunohistochemical analysis with combinative semiquantitative scoring [22]. Based on the following scale, the presence of immunopositive cells was determined: (I): 0 = no expression; 1 = weak expression; 2 = medium expression; and 3 = high expression. According to the distribution, the presence of immunopositive cells was graded as follows: (p): 1 means brown color 10%; 2 = brown > 10%; and 3 = brown > 50%. In order to determine the final score of TGF-1 immunopositive cells, the (p) score and (I) score were multiplied using the following classification: a score of 0 indicates no expression, while a score of 1–2 indicates a weakly positive expression, and a score of 3–6 indicates a strongly positive expression.

**Figure 6:**
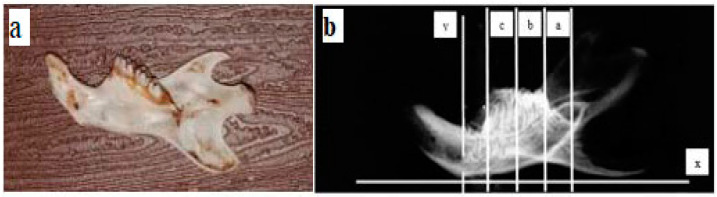
ROI of the dental socket (a) proximal area, (b) medial area (c) distal area. The plane (x) is a parallel plane to the mandibular plane and the plane (y) is a perpendicular plane to the mandibular plane.

The new bone formation was examined by measuring the area of woven bone formed in the dental socket using the image raster 3 program by calculating the area of woven bone formed in three areas that cover area a, b, and c in the region of interest (ROI) (Figure 6).

### 2.6. Statistical Analysis

TRAP, ALP, and OCN expression data were extracted using the Kruskall–Wallis test followed by the Mann–Whitney test. New bone formation data were extracted with the Saphiro Wilk test to observe the distribution of the data. If the data were normally distributed, testing was then performed using two-way ANOVA followed by the least significant difference test. A significant difference was obtained with a *p*-value of <0.05. Statistical analysis was carried out using SPSS 21.

## 3. Results

### 3.1. TRAP Expression

**Figure 7:**
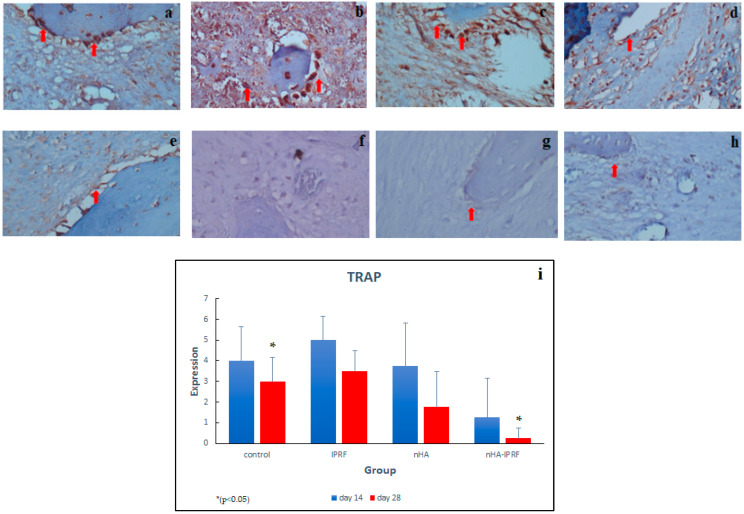
Immunohistochemical description of TRAP expression was detected as a brown color (red arrow) at 400× magnification. (**a**) Control group on day 14, (**b**) control group on day 28, (**c**) group IPRF on day 14, (**d**) group I-PRF on day 28, (**e**) nHA group day on 14, (**f**) nHA group on day 28, (**g**) nHA-IPRF group on day 14, (**h**) nHA-IPRF group on day 28, and (**i**) TRAP expression graph in each group. The control group on day 28 showed a significant difference from the nHA-IPRF group on the same day (*p* = 0.017).

The distribution of TRAP in the control, IPRF, and nHA groups on day 14 was rather high, while in the nHA group, this only occurred on day 28, while it was low in the nHA-IPRF group on day 14 and 28 (Figure 7a–h). The implantation of combined nHA and IPRF affected the expression of TRAP in bone healing after tooth extraction, where there was a significant difference between the nHA-IPRF group and the control group on day 28. The Kruskall–Wallis test followed by the Mann–Whitney U test revealed a significant difference between the control group on day 28 and the nHA-IPRF group on the same day.

### 3.2. ALP Expression

**Figure 8:**
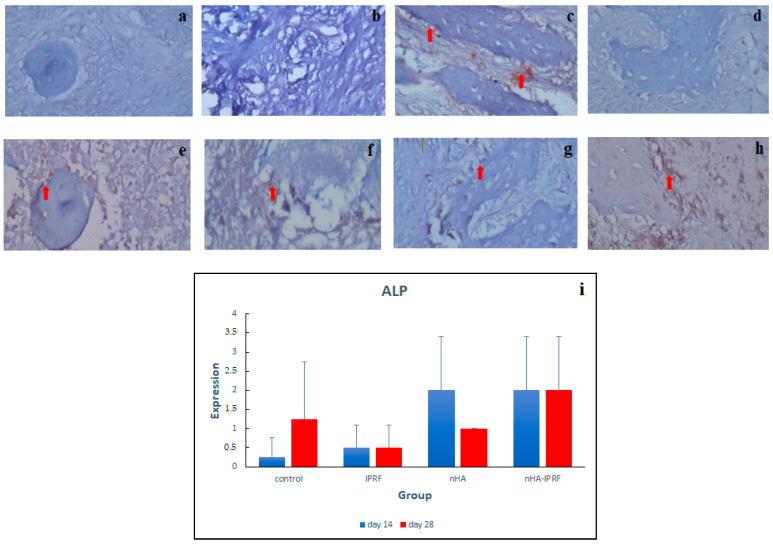
Immunohistochemical description of ALP expression was detected as a brown color (red arrow) at 400× magnification. (**a**) Control on day 14, (**b**) control on day 28, (**c**) IPRF on day 14, (**d**) IPRF on day 28, (**e**) nHA on day 14, (**f**) nHA on day 28, (**g**) nHA-IPRF on day 14, (**h**) nHA- IPRF on day 28, (**i**) and ALP expression graph in each group. There was an increase in ALP in the nHA-IPRF group on day 14 and 28 compared with the control group on the same days, although it was not statistically significant.

It was seen that the intensity and distribution of ALP in the control group and IPRF were low (Figure 8a–h). The intensity and distribution of ALP in the nHA and nHA-IPRF groups were found to be high. The results showed high ALP expression on day 14 and 28 in the nHA-IPRF combination group compared with the control group, although it was not statistically significant (Figure 8i).

### 3.3. OCN Expression

**Figure 9:**
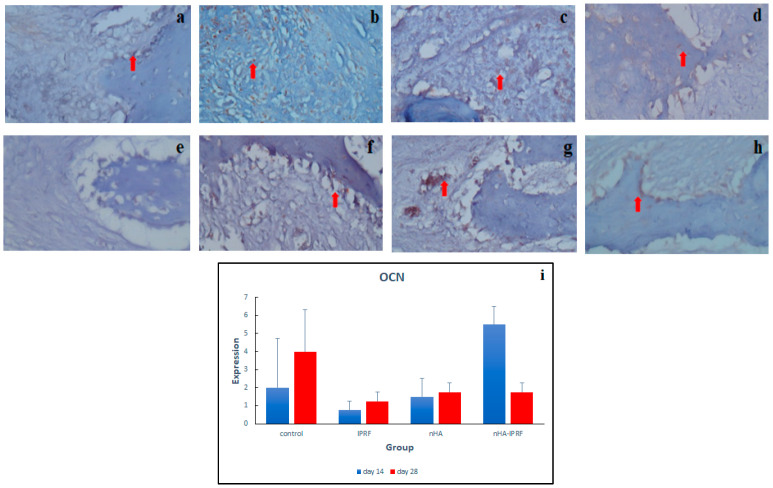
Immunohistochemical description of OCN expression were detected as a brown color (red arrow) at 400× magnification. (**a**) Control group on day 14, (**b**) control group on day 28, (**c**) IPRF group on day 14, (**d**) IPRF group on day 28, (**e**) group nHA on day 14, (**f**) group nHA on day 28, (**g**) group nHa-IPRF on day 14, (**h**) group nHa-IPRF on day 28, and (**i**) OCN expression graph in each group. There was an increase in OCN in the nHA-IPRF group on day 14 compared with the control group on the same day, although it was not statistically significant.

The results of this study pointed out that OCN expression in the nHA-IPRF group was higher than that in the control group on day 14. The trend of increased OCN expression from day 14 to day 28 was also seen in the IPRF group and nHA group (Figure 9a–h). OCN expression in the nHA-IPRF combination group was lower than in the control group on day 28 (Figure 9i).

### 3.4. New Bone Formation in Post-Extraction Rat Tooth Socket

**Figure 10:**
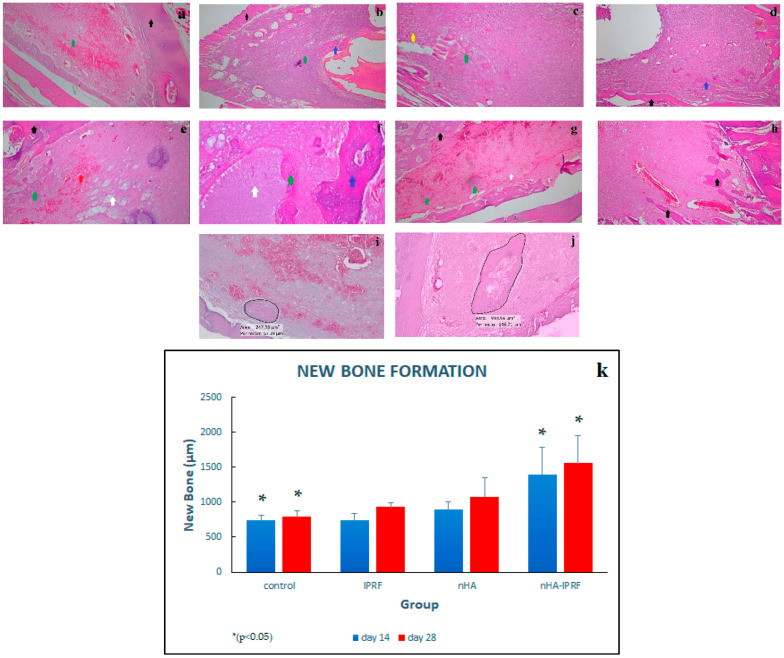
New bone formation description with hematoxilin/eosin (HE) staining. Base of extraction socket (black arrow), woven bone (green arrow), mature bone (blue arrow), nHA (white arrow), lymphocites (yellow arrow), (**a**) Control group on day 14, (**b**) control group on day 28, (**c**) IPRF group on day 14, (**d**) IPRF group on day 28, (**e**) nHa group on day 14, (**f**) nHA group on day 28, (**g**) nHA-IPRF group on day 14, and (**h**) nHA-IPRF group on day 28. (**i**) Calculation of new bone area in the control group on day 14, (**j**) calculation of new bone area in the nHA-IPRF group on day 14, and (**k**) new bone formation graph in each group. The control group on day 14 showed a significant difference from the nHA-IPRF group on the same day; likewise, the control group on day 28 showed a significant difference from the nHA-IPRF group on the same day.

It was seen that new bone formation was the highest in the nHA-IPRF group and the lowest in the control group (Figure 10a–h). The two-way ANOVA test followed by the LSD test showed significant differences between the control group on day 14 and nHA-IPRF group on the same day, as well as the control group on day 28 and nHA-IPRF group on the same day (Figure 10i).

## 4. Discussion

Alveolar preservation with bone graft increases and accelerates bone healing and prevents excessive resorption of alveolar bone. Therefore, various bone graft materials were developed in the form of either a single or combined material. The combination of nHA with biological biomaterials has been widely used to improve and accelerate tissue regeneration. A review systematic study by Zaffarin et al. (2021) concluded that nHA was very effective as a delivery system for bone regeneration and binds to proteins, drugs such as antibiotics, and other active molecules, thereby increasing osteogenesis in vivo [23]. nHA has bone mineral and good biodegradability properties. It also allows proteins, drugs, and active molecules to attach and slowly be released in bone. nHA has a particle diameter size of less than 100 nm, which makes it efficiently internalized and means that it can have an excellent surface area for cell attachment, drugs, and other bioactive molecules. This study used nHA made from a pensi shell (*corbicula moltkiana*) as well as IPRF in combination with nHA to produce a synergistic combination, and found that it can increase bone regeneration ability. IPRF is a platelet concentrate with advantages over the previous generation because it is obtained from liquid with a low speed centrifugation, making it easier to mix with other materials [24].

The results of this study proved that the implantation of the nHA-IPRF combination can increase and accelerate new bone formation and inhibit alveolar bone resorption after tooth extraction, as indicated by several biomarkers, such as decreased expression of TRAP, as the indicator of resorption, and increased expression of ALP and OCN, as the indicator of bone formation. Day 14 and 28 were selected as the time points in this study. This selection was based on the fact that, on day 14, the bone healing process entered the proliferative phase, where woven bone formation had started to form, while on day 28, the bone healing process entered a remodeling period, where the growth of woven bone was able to fill the tooth socket [3,25].

The results reported that the implantation of combined nHA and IPRF affected the expression of TRAP in bone healing after tooth extraction, where there was a significant difference between the nHA-IPRF group and the control group on day 28 (Figure 7). The mean difference in TRAP expression between the nHA-IPRF group and the control group was also seen on day 14, although it was not statistically significant. This study revealed that TRAP expression on days 14 and 28 was likely to be lower in the group implanted with the nHA-IPRF combination compared with the control group, where the post-extraction dental socket was not implanted with any material and left to fill with blood clots. This presumably occurred because the nHA-IPRF implantation controls excessive resorption of alveolar bone, as indicated by lower TRAP expression in the nHA-IPRF group.

TRAP has been widely known to play a role in signaling the active bone resorption process and as a marker of osteoclast activity. A decrease in TRAP expression indicates reduced bone resorption. Alhasimi et al. (2018) made similar suggestions on the combination of carbonate hydroxyapatite with aPRF, where the combination of these ingredients can improve post-orthodontic treatment stabilization, marked by increased osteoblasts, decreased osteoclast, and TRAP [26]. Ayukawa et al. (2009) disclosed that local application of statins can improve bone healing through osteoclast suppression and increased osteoblasts in rat bone healing areas. In this study, simvastatin was found to have affected the decrease in TRAP [27]. Boaini et al. (2018) demonstrated that a coating of strontium hydroxyapatite material on titanium material can inhibit osteoclasgenesis and osteoclast differentiation, shown through a decreased number of TRAP [28].

The results showed high ALP expression on day 14 and 28 in the nHA-IPRF combination group compared with the control group, although it was not statistically significant (Figure 8). This finding indicates that the nHA-IPRF combination biomaterial is very good at stimulating bone growth. ALP is known to be a specific glycoprotein involved in the early stages of osteoblast differentiation and is responsible for the formation of hydroxyapatite crystals in the early days of bone formation [29].

The results of this study pointed out that OCN expression in the nHA-IPRF group was higher than that in the control group on day 14 (Figure 9). Hassumi et al. (2018) argued that increased OCN expression indicates bone organization and maturation [30]. The trend of increased OCN expression from day 14 to day 28 was also seen in the IPRF group and nHA group. OCN expression in the nHA-IPRF combination group was lower than in the control group on day 28, which indicates that bone organization and maturation in the nHA-IPRF group first occurred on day 14, reached its peak, and then declined on day 28. The results of this study corresponded with those of Damayanti et al. (2020), where osteocalcin expression was higher in the implantation of combined hydroxyapatite and PRF than that of combined hydroxyapatite and platelet rich plasma on days 3, 7, and 14 in rabbit tooth post-extraction healing [31].

Important phenomena were seen in nHA group as nHA material shows the ability to increase osteogenesis by facilitating osteoblast cells and new blood vessels to develop to form a new bone, among which are those found in Figure 10e, where there was growth of woven bone and new blood vessels around the nHA material on day 14, while on day 28 (Figure 10f), woven bone was formed, which calcifies and mineralizes into mature bone. The results of this study also proved that, when implanted in the socket after rat tooth extraction, nHA was not cytotoxic and did not lead to excessive immunological reactions. This finding corresponds with the literature review by Bayani et al. (2017), suggesting that nHA is known as a biomaterial that is biocompatible, bioactive, bioresorbable, non-toxic, and does not lead to excessive immunological and inflammatory reactions [32]. nHA is also involved in suppressing osteoclast work, so as to minimize the resorption process, increase osteoblast differentiation and proliferation, and increase bone formation. However, the results of the study by Rothamel, et al. (2008) showed a different finding. The study found that the use of nHA for the preservation of alveolar bone after tooth extraction did not generate effects that could prevent changes in alveolar bone dimensions [33].

The results also showed that the IPRF group on day 14 indicates the accumulation of lymphocytes, plasma cells, and histiocytes in the post-extraction dental socket (Figure 10c), which are allegedly the effects of IPRF application, which contains leukocytes and cytokines. IPRF content makes it anti-bacterial and anti-inflammatory, although the accumulation of lymphocytes, plasma cells, and histiocytes was no longer found in the IPRF group on day 28. The results of this study were consistent with those of Varela et al. (2018), which proved that IPRF has a significantly more lymphocyte composition than blood. Furthermore, IPRF is known to contain platelets, leukocytes, type 1 collagen, osteocalcin, and growth factors [34].

The results showed that new bone growth was formed in all groups, with the highest occurring in the nHA-IPRF combination group, while the lowest occurred in the control group on both day 14 and day 28. Figure 10 demonstrates the histological description of the dental socket in the control group, which shows less new bone growth than the other group on day 14, and the nHA-IPRF group shows the greatest new bone formation, while mature bone formation began to appear on day 28 in all groups.

Statistical analysis using two-way ANOVA indicates significant differences between the groups, thus it was followed by the LSD posthoc test. The test found that there was a significant difference between the nHA-IPRF combination group and the control group on both day 14 and day 28. This finding proves that implantation of the nHA-IPRF combination can increase new bone growth on day 14 and day 28.

New bone growth upon implantation of the nHA-IPRF combination increased because nHA is known as a biocompatible, osteoconductive, and bioactive material that can foster bone growth, while IPRF contains platelet cells, leukocytes, and growth factors that can stimulate bone healing. The composition of nHA and IPRF was found to contribute considerably to increasing new bone growth, where new bone growth in the nHA group and IPRF group was higher than in the control group on both day 14 and day 28, although it was not statistically significant. The ability of nHA to increase bone regeneration is crucial. Hydroxyapatite will release calcium phosphate when hydroxyapatite is implanted into bone defects. The activity will increase body fluid saturation and precipitate biological apatite in the area. This biological apathy can contain endogenous proteins and act as a matrix for attachment and growth of osteogenic cells [13]. IPRF contributes considerably to the large increase in new bone formation by the nHA-IPRF group, because it contains growth factors, which includes platelet-derived growth factor (PDGF), transforming growth factor (TGF), and insulin growth factor (IGF), involved in the process of osteogenesis [35,36].

This study has disclosed that the combination of nHA and IPRF can increase bone regeneration and reduce alveolar bone resorption following rat tooth extraction; therefore, it is potentially a material for alveolar bone preservation. The results agree with those of Mu et al. (2020), which proved that the combination of deproteinized bovine bone mineral with IPRF accelerated vascular formation, bone remodeling, and replacement of bone graft material with new bone in the early stages of healing, despite that the combination of materials did not show an increase in bone volume at the long-term healing stage [37]. The study by Wang et al. (2021) proved that the combination of particulate bone substitute (Bio-oss) with IPRF has a positive effect on the thickness of the labial part of hard tissue in the coronal implant 6 months postoperatively [38]. Kyyak et al. (2021) also revealed that the combination of bovine bone substitute materials with IPRF increases the viability and metabolic activity of human osteoblasts, as well as increases the expression of ALP, BMP-2 at the initial level, and OCN at the final level in vitro [39]. Mallappa et al., (2022) disclosed that the combination of nHA, aPRF, and IPRF materials shows good clinical and radiological results in the treatment of intrabone periodontal defects in humans compared with the single use of nHA [40].

Based on the results of this study, the combination of nHA and IPRF has the potential to be developed into good bone graft material for alveolar bone preservation. On the other hand, the results of this study have a number of limitations, such as the small number of samples, limited observation period, and limitations in controlling the bias potential due to confounding variables. The study period consists of day 14 and day 28 only. As a result, the healing process can only be assessed on the two days, while the healing process took place throughout the study, and some healing biomarkers can reach peak fluctuations in different periods. Observations were not taken until the complete recovery of post-extraction tooth socket. The future study is expected to involve a larger number of samples as well as a longer period of observation to evaluate the total recovery of the alveolar bone after nHA-IPRF administration.

## 5. Conclusions

The nHA-IPRF combination can suppress alveolar bone resorption, marked by decreased TRAP expression, and can increase bone growth, as indicated by an increase in ALP expression, OCN, and new bone formation. nHA-IPRF implantation is feasible as a bone graft material for alveolar bone preservation because it can potentially reduce alveolar bone resorption and increase or accelerate new bone formation.

## Data Availability

The data (figures and tables) used to support the findings of this study are included within the article.

## References

[1] Ramalingam S., Sundar C., Jansen J.A., Alghamdi H. (2019). Alveolar bone science: Structural characteristics and pathological changes. Dental Implants and Bone Grafts: Materials and Biological Issues.

[2] de Sousa C.A., Lemos C.A.A., Santiago-Júnior J.F., Faverani L.P., Pellizzer E.P. (2018). Bone augmentation using autogenous bone versus biomaterial in the posterior region of atrophic mandibles: A systematic review and meta-analysis. J. Dent..

[3] Sculean A., Stavropoulos A., Bosshardt D.D. (2019). Self-regenerative capacity of intra-oral bone defects. J. Clin. Periodontol..

[4] Stumbras A., Kuliesius P., Januzis G., Juodzbalys G. (2019). Alveolar Ridge Preservation after Tooth Extraction Using Different Bone Graft Materials and Autologous Platelet Concentrates: A Systematic Review. J. Oral Maxillofac. Res..

[5] Wang C., Yu S., Fretwurst T., Larsson L., Sugai J., Oh J., Lehner K., Jin Q., Giannobile W. (2020). Maresin 1 Promotes Wound Healing and Socket Bone Regeneration for Alveolar Ridge Preservation. J. Dent. Res..

[6] Suárez F., Amo L.D., Monje A. (2022). 2022—Su rez-L pez del Amo—Efficacy of biologics for alveolar ridge preservation.pdf. J. Periodontol..

[7] Avila-Ortiz G., Gubler M., Romero-Bustillos M., Nicholas C.L., Zimmerman M.B., Barwacz C.A. (2020). Efficacy of Alveolar Ridge Preservation: A Randomized Controlled Trial. J. Dent. Res..

[8] Canellas J., Ritto F., Figueredo C., Fischer R., de Oliveira G., Thole A., Medeiros P.J. (2020). Histomorphometric evaluation of different grafting materials used for alveolar ridge preservation: A systematic review and network meta-analysis. Int. J. Oral Maxillofac. Surg..

[9] Farshidfar N., Jafarpour D., Firoozi P., Sahmeddini S., Hamedani S., de Souza R.F., Tayebi L. (2022). The application of injectable platelet-rich fibrin in regenerative dentistry: A systematic scoping review of In vitro and In vivo studies. Jpn. Dent. Sci. Rev..

[10] Khanijou M., Seriwatanachai D., Boonsiriseth K., Suphangul S., Pairuchvej V., Srisatjaluk R.L., Wongsirichat N. (2018). Bone graft material derived from extracted tooth: A review literature. J. Oral Maxillofac. Surgery, Med. Pathol..

[11] Zhou M., Geng Y.-M., Li S.-Y., Yang X.-B., Che Y.-J., Pathak J.L., Wu G. (2019). Nanocrystalline Hydroxyapatite-Based Scaffold Adsorbs and Gives Sustained Release of Osteoinductive Growth Factor and Facilitates Bone Regeneration in Mice Ectopic Model. J. Nanomater..

[12] Fukuba S., Okada M., Nohara K., Iwata T. (2021). Alloplastic Bone Substitutes for Periodontal and Bone Regeneration in Dentistry: Current Status and Prospects. Materials.

[13] Calasans-Maia M.D., Barboza Junior C.A.B., Soriano-Souza C.A., Alves A.T.N.N., Uzeda M.J.D.P., Martinez-Zelaya V.R., Mavropoulos E., Rocha Leão M.H., de Santana R.B., Granjeiro J.M. (2019). Microspheres of alginate encapsulated minocycline-loaded nanocrystalline carbonated hydroxyapatite: Therapeutic potential and effects on bone regeneration. Int. J. Nanomed..

[14] Mu Z., Chen K., Yuan S., Li Y., Huang Y., Wang C., Zhang Y., Liu W., Luo W., Liang P. (2020). Gelatin Nanoparticle-Injectable Platelet-Rich Fibrin Double Network Hydrogels with Local Adaptability and Bioactivity for Enhanced Osteogenesis. Adv. Heal. Mater..

[15] Shokry M., Mohamed S.I., Ismail R., Alshaimaa Shabaan A. (2022). The effect of Nanocrystalline Hydroxyapatite-parathyroid hormone mixture on Bone Defect Healing: Experimental Study in Dogs. Egypt. Dent. J..

[16] Xu G., Shen C., Lin H., Zhou J., Wang T., Wan B., Binshabaib M., Forouzanfar T., Xu G., Alharbi N. (2022). Development, In-Vitro Characterization and In-Vivo Osteoinductive Efficacy of a Novel Biomimetically-Precipitated Nanocrystalline Calcium Phosphate With Internally-Incorporated Bone Morphogenetic Protein-2. Front. Bioeng. Biotechnol..

[17] Albeshri S., Albialhess A., Niazy A.A., Ramallngam S., Sundar C., Alghamdi H.S. (2018). Biomarkers as Independent Predictors of Bone Regeneration around Biomaterials: A Systematic Review of Literature. J. Contemp. Dent. Pract..

[18] Azis Y., Jamarun N., Arief S., Nur H. (2015). Facile Synthesis of Hydroxyapatite Particles from Cockle Shells (Anadaragranosa) by Hydrothermal Method. Orient. J. Chem..

[19] Labanni A., Zulhadjri, Handayani D., Ohya Y., Arief S. (2020). Size controlled synthesis of well-distributed nano-silver on hydroxyapatite using alkanolamine compounds. Ceram. Int..

[20] Karde P.A., Sethi K sunder Mahale S.A., Khedkar S.U., Patil A.G., Joshi C.P. (2020). Comparative evaluation of platelet count and antimicrobial effi cacy of injectable platelet-rich fi brin with other platelet concentrates: An in vitro study. J. Indian Soc. Periodontol..

[21] Rakhmatia Y.D., Ayukawa Y., Furuhashi A., Koyano K. (2018). Carbonate Apatite Containing Statin Enhances Bone Formation in Healing Incisal Extraction Sockets in Rats. Materials.

[22] Fedchenko N., Reifenrath J. (2014). Different approaches for interpretation and reporting of immunohistochemistry analysis results in the bone tissue—A review. Diagn. Pathol..

[23] Zaffarin A.S.M., Ng S.-F., Ng M.H., Hassan H., Alias E. (2021). Nano-Hydroxyapatite as a Delivery System for Promoting Bone Regeneration In Vivo: A Systematic Review. Nanomaterials.

[24] Thanasrisuebwong P., Surarit R., Bencharit S., Ruangsawasdi N. (2019). Influence of Fractionation Methods on Physical and Biological Properties of Injectable Platelet-Rich Fibrin: An Exploratory Study. Int. J. Mol. Sci..

[25] Gomes P.D.S., Daugela P., Poskevicius L., Mariano L., Fernandes M.H. (2019). Molecular and Cellular Aspects of Socket Healing in the Absence and Presence of Graft Materials and Autologous Platelet Concentrates: A Focused Review. J. Oral Maxillofac. Res..

[26] Alhasyimi A.A., Pudyani P.P., Asmara W., Ana I.D. (2018). Enhancement of post-orthodontic tooth stability by carbonated hydroxyapatite-incorporated advanced platelet-rich fibrin in rabbits. Orthod. Craniofac. Res..

[27] Ayukawa Y., Yasukawa E., Moriyama Y., Ogino Y., Wada H., Atsuta I., Koyano K. (2009). Local application of statin promotes bone repair through the suppression of osteoclasts and the enhancement of osteoblasts at bone-healing sites in rats. Oral Surgery, Oral Med. Oral Pathol. Oral Radiol. Endodontology.

[28] Boanini E., Torricelli P., Sima F., Axente E., Fini M., Mihailescu I.N., Bigi A. (2018). Gradient coatings of strontium hydroxyapatite/zinc β-tricalcium phosphate as a tool to modulate osteoblast/osteoclast response. J. Inorg. Biochem..

[29] Rodrigues W.C., Fabris A.L.D.S., Hassumi J.S., Gonçalves A., Sonoda C.K., Okamoto R. (2016). Kinetics of gene expression of alkaline phosphatase during healing of alveolar bone in rats. Br. J. Oral Maxillofac. Surg..

[30] Hassumi J.S., Mulinari-Santos G., Fabris A.L.D.S., Jacob R.G.M., Gonçalves A., Rossi A.C., Freire A.R., Faverani L.P., Okamoto R. (2018). Alveolar bone healing in rats: Micro-CT, immunohistochemical and molecular analysis. J. Appl. Oral Sci..

[31] Damayanti M.M., Hernowo B.S., Susanah S. (2020). Osteocalcin expression of platelet-rich fibrin (PRF) and platelet-rich plasma (PRP) added with hydroxyapatite (HA) in rabbit’s post extraction tooth sockets. Padjadjaran J. Dent..

[32] Bayani M., Torabi S., Shahnaz A., Pourali M. (2017). Main properties of nanocrystalline hydroxyapatite as a bone graft material in treatment of periodontal defects. A review of literature. Biotechnol. Biotechnol. Equip..

[33] Rothamel D., Schwarz F., Herten M., Engelhardt E., Donath K., Kuehn P., Becker J. (2008). Dimensional ridge alterations following socket preservation using a nanocrystalline hydroxyapatite paste. A histomorphometrical study in dogs. Int. J. Oral Maxillofac. Surg..

[34] Varela H.A., Oliveira M.A.P.P.N., Pereira J., Souza J.C.M., Pinto N., Quirynen M. (2018). Platelet-rich fibrin to incorporate bioactive graft materials. Nanostructured Biomaterials for Cranio-Maxillofacial and Oral Applications.

[35] Du M., Chen J., Liu K., Xing H., Song C. (2021). Recent advances in biomedical engineering of nano-hydroxyapatite including dentistry, cancer treatment and bone repair. Compos. Part B Eng..

[36] Liu Z., Jin H., Xie Q., Jiang Z., Guo S., Li Y., Zhang B. (2019). Controlled Release Strategies for the Combination of Fresh and Lyophilized Platelet-Rich Fibrin on Bone Tissue Regeneration. BioMed Res. Int..

[37] Mu Z., He Q., Xin L., Li Y., Yuan S., Zou H., Shu L., Song J., Huang Y., Chen T. (2020). Effects of injectable platelet rich fibrin on bone remodeling in combination with DBBM in maxillary sinus elevation: A randomized preclinical study. Am. J. Transl. Res..

[38] Wang M., Zhang X., Li Y., Mo A. (2021). Lateral Ridge Augmentation with Guided Bone Regeneration Using Particulate Bone Substitutes and Injectable Platelet-Rich Fibrin in a Digital Workflow: 6 Month Results of a Prospective Cohort Study Based on Cone-Beam Computed Tomography Data. Materials.

[39] Kyyak S., Blatt S., Schiegnitz E., Heimes D., Staedt H., Thiem D.G.E., Sagheb K., Al-Nawas B., Kämmerer P.W. (2021). Activation of Human Osteoblasts via Different Bovine Bone Substitute Materials With and Without Injectable Platelet Rich Fibrin in vitro. Front. Bioeng. Biotechnol..

[40] Mallappa J., Vasanth D., Gowda T.M., Shah R., Gayathri G.V., Mehta D.S. (2022). Clinicoradiographic evaluation of advanced-platelet rich fibrin block (A PRF + i PRF + nanohydroxyapatite) compared to nanohydroxyapatite alone in the management of periodontal intrabony defects. J. Indian Soc. Periodontol..

